# Parallel pathways in the biosynthesis of aminoglycoside antibiotics

**DOI:** 10.12688/f1000research.11104.1

**Published:** 2017-05-18

**Authors:** Yi Yu, Qi Zhang, Zixin Deng

**Affiliations:** 1 Key Laboratory of Combinatorial Biosynthesis and Drug Discovery (Ministry of Education), School of Pharmaceutical Sciences, Wuhan University, 185 East Lake Road, Wuhan 430071, China; 2 Department of Chemistry, Fudan University, Shanghai 200433, China

**Keywords:** aminoglycoside, antibiotics, Resistance

## Abstract

Despite their inherent toxicity and the global spread of bacterial resistance, aminoglycosides (AGs), an old class of microbial drugs, remain a valuable component of the antibiotic arsenal. Recent studies have continued to reveal the fascinating biochemistry of AG biosynthesis and the rich potential in their pathway engineering. In particular, parallel pathways have been shown to be common and widespread in AG biosynthesis, highlighting nature’s ingenuity in accessing diverse natural products from a limited set of genes. In this review, we discuss the parallel biosynthetic pathways of three representative AG antibiotics—kanamycin, gentamicin, and apramycin—as well as future directions towards the discovery and development of novel AGs.

## Introduction

The discovery of streptomycin from *Streptomyces griseus* by Waksman in 1943 as a pharmaceutical legend against tuberculosis inspired massive mining of similar compounds in other bacterial species, leading to the discovery of neomycins, kanamycins, gentamicins, and a variety of other aminoglycoside (AG) antibiotics ^1– 4^. Structurally, AGs possess a featured aminocyclitol core, usually as streptamine, streptidine, or 2-deoxystreptamine (2-DOS), which is decorated by one or more amino-sugars at the C4, C5, and C6 positions. These positively charged amino groups enable AGs to interact with negatively charged molecules, especially rRNAs of bacterial 30S and 50S ribosomal subunits, thereby inhibiting protein synthesis and causing cell death ^5, 6^. Such a bactericidal property of AGs makes them broad-spectrum antibiotic agents that are effective against both Gram-negative and Gram-positive bacteria ^7^. However, as has been observed for most antibiotics, heavy usage of AGs resulted in the rapid spread of AG-resistant pathogens, which has greatly limited their clinical practice ^8^. The most common mechanism for bacterial resistance to AGs is inactivation of AGs by structural modifications ^9^. Thus far, over 100 AG-modifying enzymes (AMEs), including AG N-acetyltransferases (AACs), AG O-nucleotidyltransferases (ANTs), and AG O-phosphotransferases (APHs), have been found in a broad range of both Gram-positive and -negative organisms ^10^. Also, AG resistance can be achieved by efflux pumps and, as has been revealed recently, by 16S rRNA methyltransferase-catalyzed modifications ^11^. The bacterial resistance issues have been partly addressed by chemical modification of natural AGs to improve their pharmacological properties, as has been exemplified by the so-called second generation of AG drugs (the semisynthetic dibekacin, amikacin, netilmicin, and isepamicin) during the 1970s ^12^.

Whilst chemical engineering is an effective way to generate novel AGs, rational biosynthetic engineering of new antibiotics represents an attractive alternative, and the past decade has witnessed a massive upsurge of studies in AG biosynthesis ^13, 14^. Among the largest and best-studied subgroup of AG natural products are the 2-DOS-containing AGs ^14^. It has been well established that 2-DOS is produced from D-glucose-6-phosphate by a set of enzymes, including 2-deoxy-scyllo-inosose synthase, the dual-functional L-glutamine:2-DOI aminotransferase, and 2-deoxy-scyllo-inosamine dehydrogenase (the genes encoding these enzymes are conserved in all of the biosynthetic gene clusters identified so far for 2-DOS-containing AGs and are designated as “C, S, E”-type genes, respectively) ^7, 15, 16^. Decoration by various sugar moieties at the C4, C5, and C6 positions of 2-DOS proceeds via different patterns, yielding 4,5-disubstituted AGs (e.g. butirosin and neomycin), 4,6-disubstituted AGs (e.g. kanamycin, tobramycin, and gentamicin), or 4- and 5-monosubstituted AGs (e.g. apramycin and hygromycin). Readers are referred to many excellent reviews discussing the biosynthesis and engineering of AG antibiotics ^13– 15, 17, 18^.

A common observation in AG biosynthesis is that one producer strain usually produces many AG congeners simultaneously. Recent studies have shown that the presence of multiple AG congeners is, in many cases, not merely because of inefficient downstream modifications but mainly the result of parallel biosynthetic pathways ^19– 21^. This review discusses the recent advances in the biosynthesis of three representative AGs—kanamycin, gentamicin, and apramycin—which all involve parallel pathways. We attempt to highlight nature’s ingenuity in accessing diverse AGs through a limited set of genes and the potential for the generation of novel AG antibiotics by pathway engineering efforts.

## Parallel pathways in kanamycin biosynthesis

The kanamycin class consists of several members, most of which were isolated from *Streptomyces kanamyceticus* ^1^. Structurally, these congeners contain the same kanosamine moiety (3-amino-3-deoxy-D-glucose) at the C6 position of 2-DOS but possess different amino-sugars at the C4 position of 2-DOS (i.e. 6-amino-6-deoxy-D-glucose in kanamycin A, neosamine in kanamycin B, and D-glucosamine in kanamycin C). Several second-generation AG drugs, such as amikacin, dibekacin, and arbekacin, are produced by semi-synthetic modification of the kanamycin core skeleton ^9^, stimulating interest in deciphering kanamycin biosynthesis and developing novel kanamycin by bioengineering.

**Figure 1.**
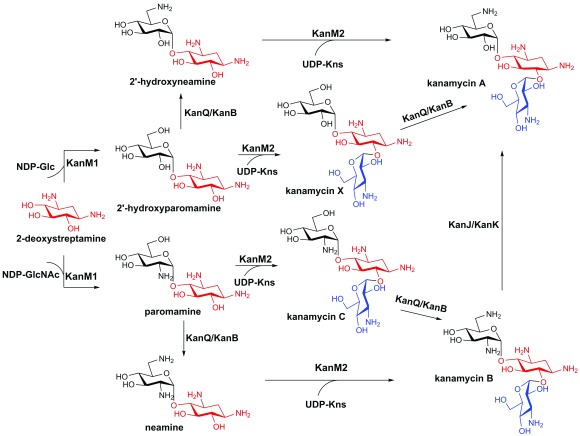
Parallel pathways in kanamycin biosynthesis. The 2-deoxystreptamine (2-DOS) moiety is highlighted in red, and the amino-sugar kanosamine (Kns) is highlighted in blue. GlcNAc, N-Acetylglucosamine.

The parallel pathways of AG biosynthesis were firstly revealed for kanamycin ^21^. For a long time, it had been postulated that kanamycin is assembled via a linear pathway ^7^. However, the recent study by Yoon, Sohng, and co-workers indicated that kanamycin A and B are actually produced by two parallel pathways ( Figure 1) ^21^. KanF (also known as KanM1), the glycosyltransferase in the *kan* gene cluster, was shown to be able to transfer both NDP-glucose and NDP-N-acetylglucosamine to 2-DOS to generate 2'-hydroxyparomamine and paromamine, respectively, thereby directing the metabolic flux of 2-DOS into two branches. The two resulting pseudodisaccharides are then aminated at the C6' position by the sequential action of a dehydrogenase KanQ (also known as KanI) and an aminotransferase KanB (also known as KacL), yielding 2'-hydroxyneamine and neamine, respectively. KanE (also known as KanM2), the second glycosyltransferase in the *kan* gene cluster, exhibits a very relaxed substrate promiscuity, accepting four different pseudodisaccharides (i.e. paromamine, neamine, 2'-hydroxyneamine, and 2'-hydroxyparomamine) as glycosyl acceptors ^21, 22^, resulting in the production of kanamycin A and B via the two independent pathways ( Figure 1). The complex network of kanamycin biosynthesis is completed by the action of the α-ketoglutarate-dependent dioxygenase KanJ and the NADH-dependent reductase KanK, which together convert kanamycin B to kanamycin A ^23^. The remarkable substrate promiscuity of kanamycin biosynthetic enzymes suggests the great potential in AG combinatorial biosynthesis. Indeed, integration of the *S*-4-amino-2-hydroxybutyric acid (AHBA) biosynthesis genes into the kanamycin pathway successfully led to the production of a semisynthetic AG, amikacin, and a new AG analogue, 1-N-AHBA-kanamycin X, both of which show improved antibacterial activity compared with kanamycin A ^21^.

## Parallel pathways in gentamicin biosynthesis

Gentamicin was isolated from *Micromonospora echinospora* as a mixture of five C-series components consisting of C1, C2, C1a, C2a, C2b ^4^. These compounds have received much attention in recent years as a broad-spectrum antibiotic for the treatment of various infections as well as a promising drug lead for the treatment of several inherited diseases and a sensitizing agent for lung cancer cells ^24– 26^. Gentamicin contains the core 2-DOS moiety with the amino-sugars purpurosamine and garosamine attached at positions C4 and C6, respectively. The biosynthetic pathway for gentamicin was firstly proposed several decades ago, which is mostly corrected as shown by recent studies ^19^. Heterologous expression of a subset of the gentamicin gene cluster in *Streptomyces venezuelae* indicated that gentamicin A2 is the first pseudotrisaccharide precursor of the gentamicin C complex ^27^. Gentamicin A2 is then converted to gentamicin X2 by a series of enzymes, including the dehydrogenase GenD2 and the pyridoxal phosphate (PLP)-dependent aminotransferase GenS2, the S-adenosylmethionine (SAM)-dependent N-methyltransferase GenN, and the radical SAM methyltransferase GenD1. GenD2 and GenS are responsible for the amination of the C3'' position of gentamicin A2, and the resulting amino group is methylated by GenN to afford a C3'' methylamino group ^28^. GenD1 belongs to the class B radical SAM methyltransferases ^29, 30^, which utilizes a cobalamin cofactor and installs a methyl group on the C4'' position ^31, 32^. Gentamicin X2 is then methylated at the C6' position by GenK, which, like GenD1, also belongs to the class B radical SAM methyltransferase, leading to the production of gentamicin G418 ^33^.

**Figure 2.**
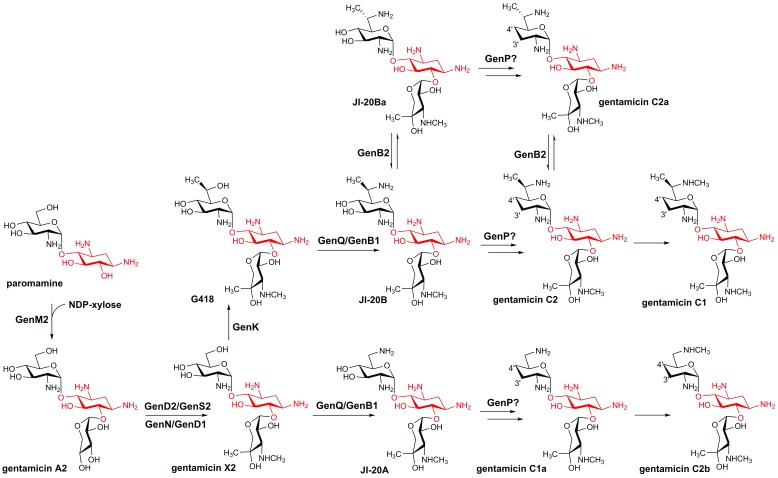
Parallel pathways in gentamicin biosynthesis. The 2-deoxystreptamine (2-DOS) moiety is highlighted in red.

Gentamicin G418 and X2 then serve as the starting substrates of the two parallel pathways and are first aminated at C6' by the sequential action of the dehydrogenase GenQ and the aminotransferase GenB1, resulting in gentamicin JI-20A and JI-20B, respectively ^19^. Gentamicin JI-20A is then dideoxygenated by a set of uncharacterized enzymes (likely including a phosphotransferase, GenP) to produce gentamicin C1a, whose C6' amino group is then methylated by an as-yet-unknown methyltransferase to produce gentamicin C2b. Gentamicin JI-20B is also dideoxygenated and methylated to afford gentamicin C1. However, because of the presence of several gentamicin congeners, including gentamicin JI-20Ba, gentamicin C2a, and gentamicin C2, and the involvement of the epimerase GenB2 that catalyzes the stereochemical interconversion of the C6' amino group, the exact reaction order and timing in gentamicin C1 production is currently unclear. A possible scenario is shown in Figure 2, in which GenB2 is responsible for the third parallel pathway and is not essential for gentamicin C biosynthesis; this hypothesis awaits testing in future studies. Unlike the parallel pathways in kanamycin biosynthesis, in which the final product of one pathway (kanamycin B) can be converted to the final product of the second one (kanamycin A), gentamicin C2b is the ultimate product and cannot be methylated by GenK to produce gentamicin C1. Clearly, many aspects in gentamicin biosynthesis remain elusive and warrant investigations.

## Parallel pathways in apramycin biosynthesis

Apramycin is a highly modified AG containing a unique bicyclic octose moiety, which is deoxygenated at the C3 position. Although currently only licensed for veterinary practice use, apramycin represents the only known AG that is active against carbapenem-resistant Enterobacteriaceae isolates harboring the 16S rRNA MTases gene ^34^. In addition, apramycin is able to mimic the features of an incompatible plasmid, thereby re-sensitizing bacteria to conventional antibiotic treatments by causing plasmid loss *in vivo* ^35^. More importantly, in contrast to common AGs that possess substantial ototoxicity and could cause irreversible hearing loss ^36^, recent studies have shown that apramycin has less ototoxicity, further promising its pharmaceutical applications ^37^.

**Figure 3.**
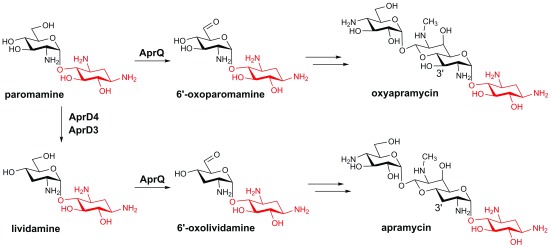
Parallel pathways in apramycin biosynthesis. The 2-deoxystreptamine (2-DOS) moiety is highlighted in red.

The biosynthetic gene cluster of apramycin was reported more than a decade ago ^38^, but the biosynthetic timing of apramycin, particularly with regard to the C3 deoxygenation step, has remained obscure until very recently. In contrast to the previous proposal in which the C3 deoxygenation occurs as the penultimate step, biochemical studies have shown that the C3 deoxygenation is catalyzed by the radical SAM diol dehydratase AprD4 and its NADPH-dependent reductase partner AprD3 ^20, 39, 40^. This C3 deoxygenation reaction does not occur on oxyapramycin but on the pseudodisaccharide substrate paromamine, demonstrating the two parallel biosynthetic pathways, which affords oxyapramycin and apramycin, respectively ( Figure 3) ^20^. Consistent with this observation, biochemical analysis has shown that AprQ, a FAD-dependent dehydrogenase, catalyzes the C6' dehydrogenation of both paromamine and lividamine, with the latter as the preferred substrate ^20^. Remarkably, AprQ is also able to act on the tripseudodisaccharide substrate gentamicin X2 and G418 and, in the latter case, produce a novel gentamicin analogue with a C6' carboxylate ^20^. A similar observation was also made on AprD4 and AprD3, which are able to act on tripseudodisaccharide substrates, such as kanamycin C and kanamycin B ^40^. The remarkable substrate promiscuity of apramycin biosynthetic enzymes demonstrates the great potential to produce novel AGs by pathway engineering efforts. It is also noteworthy that although the radical-mediated dehydration catalyzed by cobalamin-dependent diol dehydratase has been well studied, AprD4 represents the only known dehydratase in the radical SAM superfamily, and its catalytic mechanism remains to be determined.

## Perspective

In 2015, Drs Youyou Tu, William Campbell, and Satoshi Ōmura jointly received the Nobel Prize in Physiology or Medicine for their seminal discoveries of the natural products avermectin and artemisinin, partly renewing interest in natural drug discovery and development. Despite their long history of clinical and veterinary usage, AGs still represent an important class of antibiotic for the treatment of severe infections, particularly for Gram-negative bacterial infections. Because of the recent global spread of AG-resistant pathogens and the inherent drawbacks of AGs such as ototoxicity and nephrotoxicity, the discovery and development of novel AGs is in urgent need ^41, 42^. Mining of novel AGs from bacterial genomes is a promising way because of the rapid development of sequencing technology and the expansion of sequenced genomes that are publically available ( https://gold.jgi.doe.gov/) ^43^. Pathway engineering serves as another attractive strategy to generate novel and more robust AGs ^18^. The parallel biosynthetic pathways of AGs discussed above reflects nature’s wisdom in structural diversity-oriented biosynthesis, highlighting the potential to access novel and more robust AGs by combinatorial biosynthesis while taking advantage of the substrate promiscuity of AG biosynthetic enzymes. Plazomicin is a new semisynthetic AG designed to evade all clinically relevant AMEs and is currently under phase III clinical trials ^9, 44^. It contains a gentamicin-related skeleton with a hydroxy-aminobutyric acid substituent and a hydroxyethyl substituent at its N-1' and N-6' positions, respectively ^45^. The promise of plazomicin to be approved as a third-generation AG drug and its structural similarity with gentamicin will undoubtedly spark increased interest in engineering AG pathways ^46^. Given that many aspects of AG biosynthesis remain unclear, further functional and mechanistic investigation of AG biosynthetic enzymes could be rewarding, which very likely entails novel and interesting biochemistries. With the expansion of our knowledge in AG biosynthesis and the availability of more and more synthetic biological tools, the resurgence of this old class of antibiotics is possible in the foreseeable future.

## Abbreviations

AG, aminoglycoside; AHBA, S-4-amino-2-hydroxybutyric acid; AME, aminoglycoside-modifying enzyme; 2-DOS, 2-deoxystreptamine; SAM, S-adenosylmethionine.
